# An ultra-compact integrated system for brain activity recording and stimulation validated over cortical slow oscillations *in vivo* and *in vitro*

**DOI:** 10.1038/s41598-018-34560-y

**Published:** 2018-11-13

**Authors:** Luca Pazzini, Davide Polese, Julia F. Weinert, Luca Maiolo, Francesco Maita, Marco Marrani, Alessandro Pecora, Maria V. Sanchez-Vives, Guglielmo Fortunato

**Affiliations:** 1 Istituto per la Microelettronica e Microsistemi - Consiglio Nazionale delle Ricerche, Rome, Italy; 2 Institut d’Investigacions Biomèdiques August Pi i Sunyer (IDIBAPS), Barcelona, Spain; 3 ICREA, Barcelona, Spain

## Abstract

The understanding of brain processing requires monitoring and exogenous modulation of neuronal ensembles. To this end, it is critical to implement equipment that ideally provides highly accurate, low latency recording and stimulation capabilities, that is functional for different experimental preparations and that is highly compact and mobile. To address these requirements, we designed a small ultra-flexible multielectrode array and combined it with an ultra-compact electronic system. The device consists of a polyimide microelectrode array (8 µm thick and with electrodes measuring as low as 10 µm in diameter) connected to a miniaturized electronic board capable of amplifying, filtering and digitalizing neural signals and, in addition, of stimulating brain tissue. To evaluate the system, we recorded slow oscillations generated in the cerebral cortex network both from *in vitro* slices and from *in vivo* anesthetized animals, and we modulated the oscillatory pattern by means of electrical and visual stimulation. Finally, we established a preliminary closed-loop algorithm *in vitro* that exploits the low latency of the electronics (<0.5 ms), thus allowing monitoring and modulating emergent cortical activity in real time to a desired target oscillatory frequency.

## Introduction

Given the electrical nature of brain activity, electrophysiological techniques have been used to study brain behavior since Richard Caton performed the first brain recordings in the 19^th^ century^1^. Electrical recordings provide precise temporal resolution, whereas the spatial resolution varies according to the probes used. The recording and acquisition of electrical brain activity requires a system that is basically composed of three main parts: a set of sensors that capture the neural signals, an amplification and digitization system, and a communication interface connected to a computer.

The first part of the system, i.e. the electrodes constituting the interface with the brain, can vary depending on the objective of the electrophysiological recordings. They can range from invasive, depth electrodes, which can include single cell recordings, to non-invasive superficial scalp electrodes sensing large neuronal populations with low spatial resolution. It is however challenging to design probes that have good spatial resolution and high signal accuracy and that, on top of that, do not damage the brain tissue. Thanks to the progress in material science and in microfabrication techniques, many advances have been achieved in manufacturing electrodes with high spatial resolution, improved sensitivity and minimally damaging, with higher conformability and an optimized material biocompatibility. Microelectrode arrays (MEAs) used for electrocorticography (ECoG) as epicortical devices placed on the exposed surface of the cortex represent a good compromise between a low degree of invasiveness and high signal accuracy. The efficacy of the MEAs as brain probes has been demonstrated in studies that provide insights into the processing strategies of the brain^2–9^.

The second important part of the acquisition system is the readout electronics, which must be suitable for recording, amplifying and managing the signals captured by the MEAs. Currently available neural interfaces still have to improve their reliability and they generally need bulky amplification or analysis systems that are used for animal or human medical applications. Our objective was thus to design and experimentally test an ad hoc compact electronic platform that could easily acquire, amplify and digitalize data in real time. Nevertheless, this integration is not trivial; indeed, several issues had to be addressed: (*i)* the choice of a biocompatible and ultra-flexible substrate for the electrodes; (*ii)* the interfacing of this probe with electronics able to manage the incoming signals; and (*iii)* the manufacturing of a compact and portable system that can offer stimulation control and closed-loop functionality.

In this work, we devised an ultra-compact system (hereafter called Corticonic system) composed of ultra-flexible microelectrode arrays (UF-MEAs) connected to a recording and stimulation board (hereafter called Corticonic board) that fits in the palm of one hand. Even though the system can be used for a wide variety of electrophysiological applications, we conceived the system for the recording of cortical activity and we used it for the recording of slow waves in cortical slices *in vitro* and on the cortical surface (micro ECoG) *in vivo*, as well as for the recording of electrically and visually evoked activity.

Slow oscillations are a slow (<1 Hz) oscillatory activity pattern that consists of alternating active periods with neuronal firing (Up states) and silent periods (Down states) and that arises spontaneously in the cortex during non-rapid eye movement (non-REM) sleep and during deep anesthesia^10^. Slow oscillations are also expressed under other conditions such as physical disconnection of the cerebral cortex^11,12^, in clinical conditions following a traumatic or cerebrovascular disorder resulting in a “cortical island”^13^ and in cortical slices^14^. As a result, the alternation between metastable cortical Up and Down states expressed in slow oscillations has been argued to be the default cortical activity^15,16^. Within slow oscillations, Up states contain high frequencies in the beta and gamma range (15–90 Hz)^17,18^ and propagate along the cortical network as wavefronts with a speed that can reach 7 m/s in humans^14,19–21^, propagation patterns can be altered in patients with neurological diseases^17^. Different activity parameters of slow oscillations (frequency, coefficient of variation, propagation speed, gamma power, etc.) are indicative of the healthy/pathological state of the underlying network^22–24^. For all these reasons, slow oscillations represent a good electrophysiological model to test our novel system.

In addition to research on physiological brain activity, the study of the modulation of cortical activity by means of electric fields has acquired increasing relevance in the clinical realm (for a review see Brunoni *et al*.^25^), and it is also a valuable tool to investigate the cortical organization and to identify some computational principles of the network dynamics underlying the cerebral cortex^26,27^. To achieve this, we should ideally perform *in vivo* and *in vitro* stimulation and measurements with a system that is able to record and activate neural signals in large parts of the brain with high spatial and temporal accuracy. Especially in real-time stimulation and recording tests, low latencies permit controlling the cortical activity using a closed loop. To validate the properties of our electronic board in this type of modulation experiments, we tested different stimulation protocols to demonstrate preliminary closed-loop applications. Therefore, we present a novel ultra-compact electrophysiological system—the Corticonic system—composed of UF-MEAs, and a recording and stimulation board. We validated this system in slow oscillations *in vitro* and *in vivo* by comparing the performance of the Corticonic system with conventional equipment. We demonstrate that our system is a portable general purpose setup can be deployed in different biomedical application including electrocardiography, electromyography, electrocorticography and electroencephalography. The reduced dimensions, in fact, allow an easily movement and reorganization of the setup, since just a USB connection is indispensable to arrange an electrophysiological laboratory setup. We think that the better performance of the presented device respect to classical laboratory instrumentation could be crucial into detecting new brain activity details.

## Materials and Methods

### Manufacturing of the ultra-compact system

#### Microelectrode array design, fabrication and characterization

**Figure 1:**
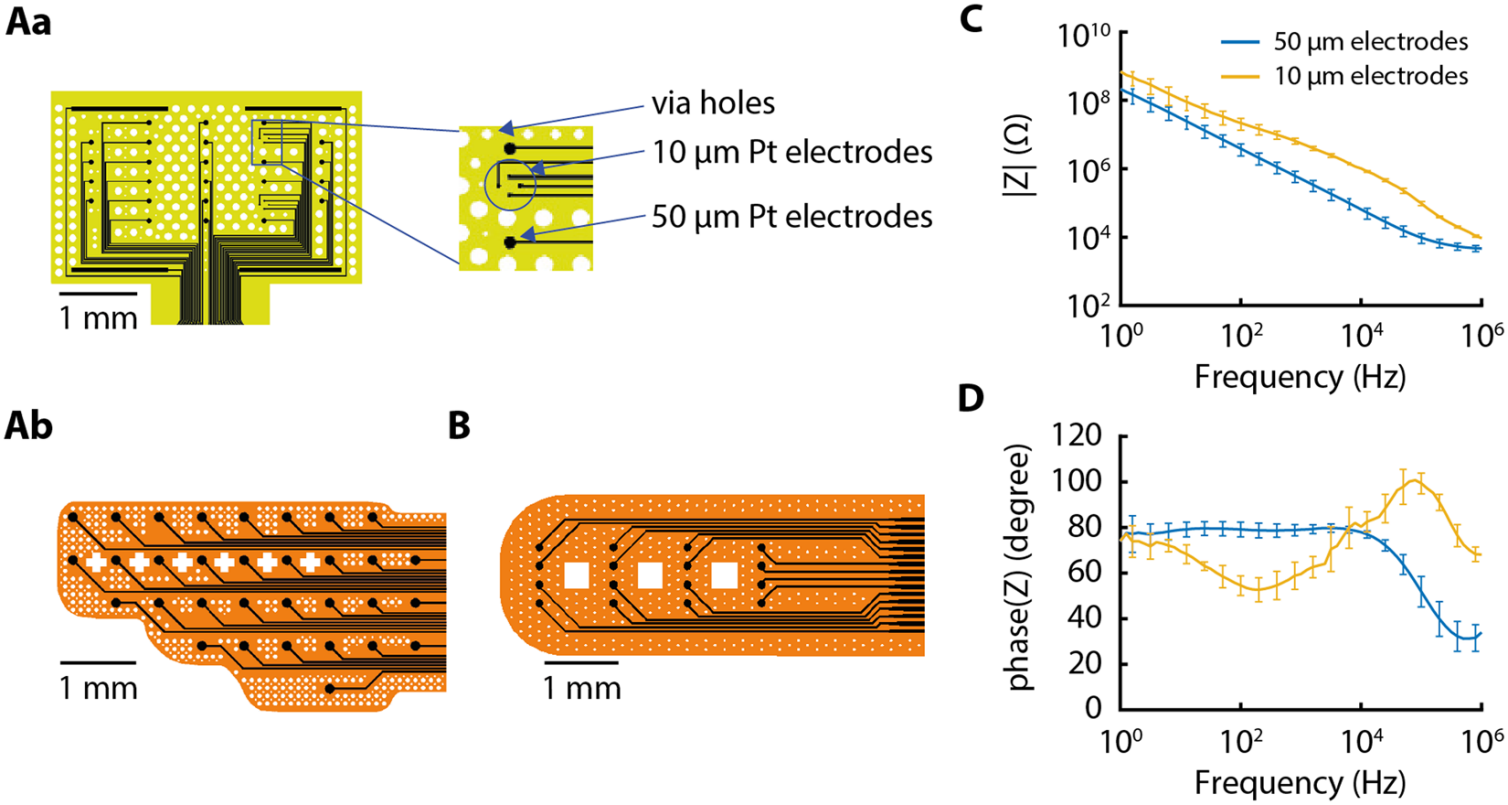
Ultra-flexible microelectrode array (UF-MEA) designs and impedance measurements. (**A**) *In vivo* UF-MEAs with 32 channels. (**Aa**) *In vivo* rectangular UF-MEA design with 24 electrodes of 50 µm in diameter and 8 electrodes in a rhomboidal arrangement of 10 µm in diameter (see inset). **(Ab**) *In vivo* hemispheric UF-MEA design fabricated to cover one full hemisphere of a mouse cortex with 32 electrodes of 50 µm in diameter. White spaces represent via holes for tissue oxygenation and better material adhesion. (**B**) *In vitro* UF-MEA design composed of 16 electrodes of 50 µm in diameter. (**C**,**D**) Bode diagrams of the impedance (Z) measurements showing (**C**) module and (**D**) phase of the electrode impedance.

According to the requirements of each specific test, different UF-MEA layouts were designed to deal with the peculiarity of *in vivo* and *in vitro* setups, hereafter called *in vivo* UF-MEAs and *in vitro* UF-MEAs (Fig. 1A,B). In particular, we produced two different *in vivo* layouts: one hemispherical (hemispherical UF-MEA) and the other with a rectangular shape and different electrode sizes for high accuracy recording (rectangular UF-MEA). In both cases, the recording sites were designed to cover several cortical areas with the aim to obtain simultaneous multisite recordings. For *in vitro* experiments, the size of the electrode grid was conceived with the intent to span all cortical layers in the slice.

UF-MEAs were fabricated by embedding a metal tri-layer of Cr/Au/Pt (200 nm thick) into polyimide (HD2611, HD MicroSystems) layers, reaching a final thickness of 8 μm^5^. The whole fabrication process was performed on an oxidized silicon wafer to guarantee better handling of the device during the several steps of the fabrication and as support for the device.

On the oxidized silicon wafer, as a first step, a 4-μm-thick polyimide layer was deposited and then thermally cured to stabilize the polymer. To improve the adhesion of the following layers on the polyimide layer, a 50-nm-thick SiN film was deposited by plasma-enhanced chemical vapor deposition (PECVD) at a temperature of 250 °C. Subsequently, a metal tri-layer (50-nm-thick Cr, 100-nm-thick Au and 50-nm–thick Pt) was evaporated on the polymeric substrate. The metal tracks were then lithographically defined, thus obtaining the electrode pads. Additionally, to passivate the devices, a further 4-μm-thick polyimide layer was spin-coated on the wafer and cured. Finally, vias were opened to recover the electrode pads by using an oxygen plasma technique in a reactive ion etching system^5^.

Furthermore, for both designs, via holes were opened in the two polyimide layers in order to provide sufficient oxygenation of the cortical slice and to achieve better surface adhesion. At the end of the fabrication process, the MEAs were mechanically detached from the rigid holder and bonded with a customized flexible printed circuit board (PCB) through a procedure that is based on anisotropic conductive film (ACF). The flexible PCB provides a standard connector to interface the MEA with the Corticonic board.

After the UF-MEA fabrication, the electrical behavior of the electrodes was evaluated by Electrochemical Impedance Spectroscopy (EIS) analysis. EIS is a widely used technique typically implemented to study the electrode impedance. In this case, we immersed the UF-MEAs in saline in order to simulate an interface similar to the brain tissue^28,29^. The impedance measurements were performed at room temperature in a Faraday cage at DC 0 V and with an AC signal of 10 mV in a stimulus range between 1 Hz and 100 kHz. The tests were carried out in NaCl solution (0.9%) in de-ionized and distilled water. The electrode pads of the UF-MEAs were individually used as working electrodes (WE) whereas an Ag/AgCl wire was used as reference electrode (RE) and a gold foil as counter electrode (CE). The measurements were performed with a VersaSTAT 4 potentiostat by PAR (Fig. 1C,D).

#### Corticonic board

**Figure 2:**
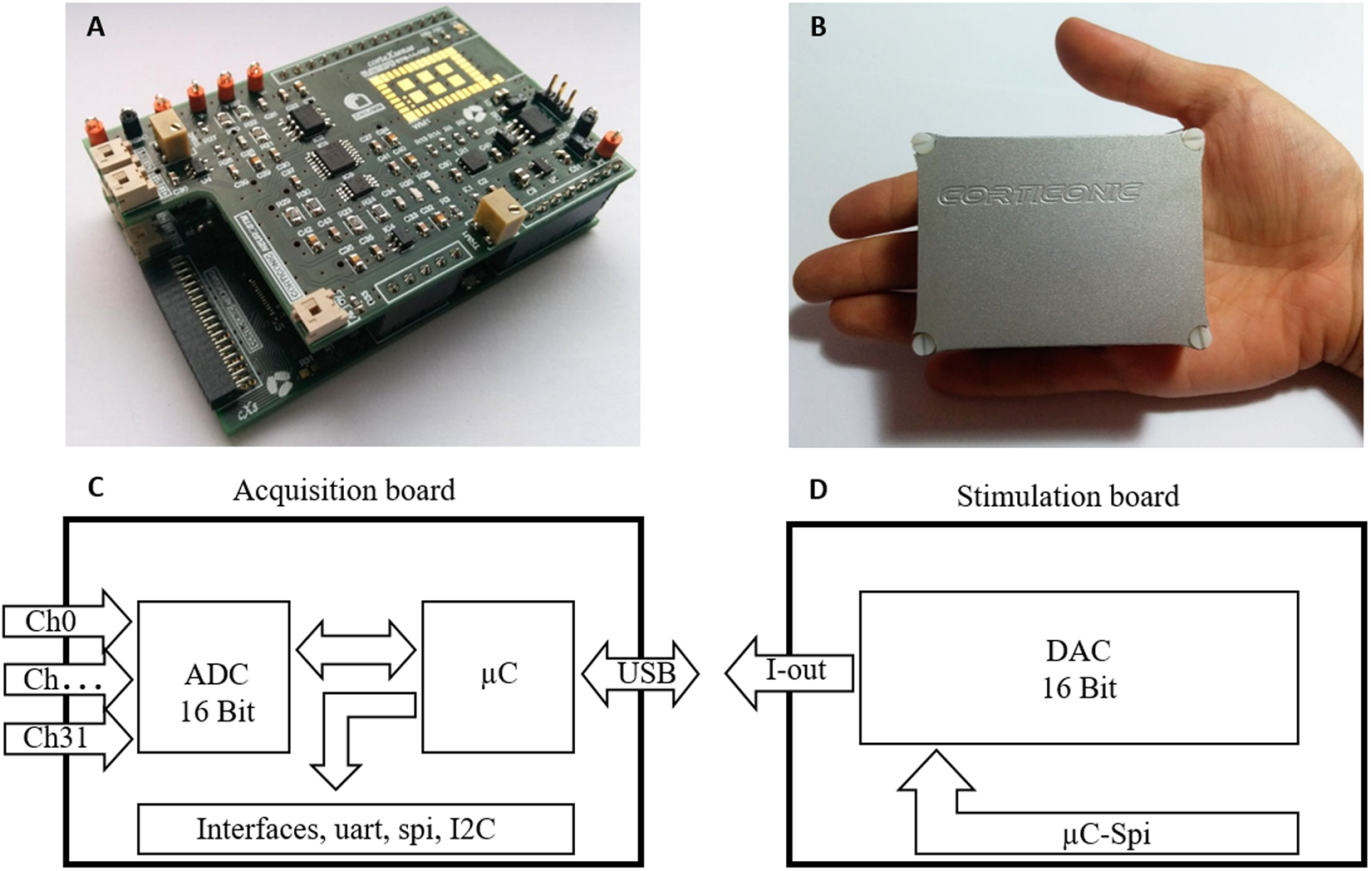
The Corticonic board. (**A**) Recording and stimulation boards connected together in a stacked configuration shown out of the shell. (**B**) Reduced dimensions of the all-in-one Corticonic board that can lie on a palm hand with its 32 recording channels and its 2 stimulation channels. (**C**) Scheme of the acquisition board with the main components (analog-to-digital converter (ADC), microcontroller (μC), Hi-Speed USB connection and Input/Output digital interfaces (I/O)). (**D**) Scheme of the stimulation board with a 16-bit digital-to-analog converter (DAC).

The ultra-compact electronic board was called Corticonic board and it is composed of two boards assembled together in a stacked configuration: one for data acquisition and, above, a second for stimulation, thus permitting simultaneous closed-loop tests of brain activity (Fig. 2A). The design strategy was focused on allowing the system to perform all the acquisition, filtering and digitalization tasks on the same compact platform. This modular configuration allows extending the system potentialities just by connecting an expansion board. Moreover, a series of different input/output (I/O) communication interfaces were added to allow further customization of the system. The Corticonic board provides a fully integrated solution that fits in the palm of one hand thanks to its reduced dimensions (Fig. 2B).

The acquisition board is composed of 32 input channels connected to an RHD2000 chip containing an amplifier and an analog-to-digital converter (ADC) by Intan technology (http://www.intantech.com), and a microcontroller (www.microchip.com) (Fig. 2C). The neuronal signals can be sampled up to 1 MSample/s and digitized at 16 bits. Moreover, the ADC is combined with a filter and a low-noise amplifier (Amplifier Input Reference Noise = 2.4 µV_rms_) that amplifies 200x. The microcontroller, in addition to managing the ADC, integrates the USB communication (High Speed USB) to permit the transfer of large amounts of data to the computer (up to 16 Mbit/s). Furthermore, the board contains several I/O and standard communication protocols for general purposes (I2C, UART, SPI) to easily control other external devices. Additionally, the acquisition board is equipped with two independent stimulation channels that enable local stimulation by using the internal stimulation board or an external source by means of AC or DC voltage and currents.

The stimulation board is composed of a dual-channel, low-noise, 16-bit digital-to-analog converter (DAC) and a dual-channel, low-noise amplifier (Fig. 2D). The stimulation board is connected to the acquisition board, which provides the power supply and controls the stimulation board by SPI from the microcontroller. The board has two independent stimulation channels that permit local stimulation in voltage or current. The outputs can be driven to generate arbitrary waveforms.

This integration of acquisition and stimulation in one system provides the possibility to easily implement and execute closed-loop algorithms.

#### Computer software

The system is controlled by a software interface developed in MATLAB (MathWorks, Natick, MA, 2000). The software guarantees easy control of several parameters for data acquisition, stimulation and closed-loop algorithms. In particular, it is possible to select the acquisition channels and the stimulation channels. Moreover, waveform shape, frequency and amplitude of the stimulation can be tuned. In train pulses stimulation, the number of repetitions and delay between consecutive trains are also selectable. Both acquisition and stimulation channels can work independently and at the same time. Additionally, parameters such as amplitude threshold, event window width and number of expected events can be controlled by software and sent to the computational unit of the board in order to set a target slow oscillation frequency.

### Validation of the UF-MEAs and Corticonic recording and stimulation system

To assess the performance of the Corticonic board and the UF-MEAs, we first characterized the impedance spectra of the UF-MEAs in saline solution, and then we contrasted the Corticonic board and the UF-MEAs with conventional equipment *in vitro* and *in vivo*, performing experiments to evaluate both recording of local field potentials (LFP) and stimulation (electrical and optical).

#### Experimental procedures

Different experiments were performed *in vivo* and *in vitro* to validate the performance of the Corticonic board and the UF-MEAs. We used a model of cortical slow oscillations (<1 Hz), which naturally occur during deep sleep and anesthesia^10^ and which can also be reproduced *in vitro* in cortical slices^30^. All the procedures were carried out in compliance with the European Community Council Directive for the care and use of laboratory animals (2010/63/EU) and with Spanish regulatory laws (BOE-A-2013-6271). All experiments were approved by the Ethics Committee of the Universitat de Barcelona.

#### *In vitro* slice preparation

**Figure 3:**
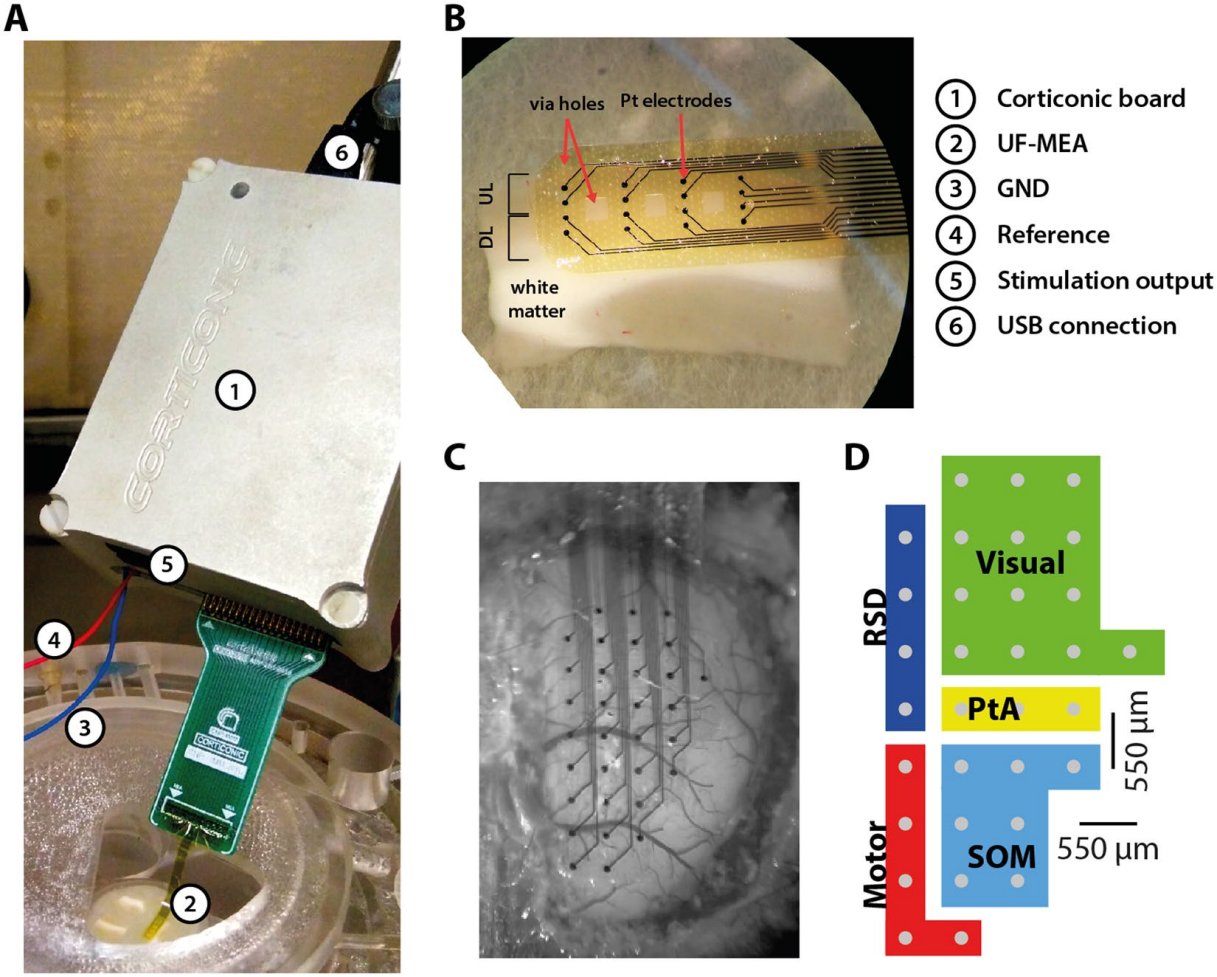
Typical experimental setup for *in vitro* and *in vivo* measurements. (**A**) Full experimental configuration of the Corticonic board (1). Connected to the board there are: the *in vitro* UF-MEA (2), the ground (3), the reference (4), the stimulation outputs (5) and the USB cable (6). (**B**) Placement of the *in vitro* UF-MEA on a cortical slice. Electrodes span across deeper and upper layers (DL and UL, respectively). Electrodes and via holes are indicated with red arrows. (**C**) *In vivo* 32-channel hemispheric UF-MEA placed on the cortex of a mouse, covering one hemisphere and spanning across different cortical regions. (**D**) Areas over which the array lays on while on the mouse cortex: PtA, parietal association; RSD, retrosplenial dysgranular cortex; SOM, somatosensory.

For *in vitro* experiments, male ferret (4–8 months) cortical slices that displayed spontaneous slow oscillatory activity (<1 Hz) were obtained as previously described^30^. Briefly, ferrets were anesthetized with pentobarbital (40 mg/kg, i.p.) and decapitated. The brain was removed and placed in an ice-cold sucrose solution^31^ and 400-µm-thick slices of the visual cortex were cut. Slices were then placed in an interface style recording chamber (Fine Science Tools) and superfused with a mixture containing 50% sucrose solution and 50% artificial cerebrospinal fluid (ACSF). ACSF contained (in mM): NaCl, 126; KCl, 2.5; MgSO_4_, 2; NaH_2_PO_4_, 1; CaCl_2_, 2; NaHCO_3_, 26; dextrose, 10. After 15 min, the slices were bathed in 100% ACSF for at least 1.5 h. Recordings were performed under modified ACSF conditions^14^. Modified ACSF was composed of the same concentrations as ACSF except for (in mM): KCl 4; MgSO_4_, 1; and CaCl_2_, 1. During the entire experiment, carbogene (95% oxygen, 5% carbon dioxide) was added to the solutions to maintain a stable pH of 7.4. Temperature was kept at 34.5–36 °C. The experimental setup and the arrangement of the MEA on the cortical slices are shown in Fig. 3A,B. Recordings started once the slices spontaneously displayed slow oscillations.

#### *In vivo* experiments

*In vivo* experiments were performed on mice (male, ~3 months, BJ-57C) under isoflurane anesthesia (0.8%). Atropine (0.3 mg/kg) was additionally delivered subcutaneously to avoid respiratory secretion. The anesthetized animals were placed in a stereotactic system and a craniotomy was performed. The skull was opened above one hemisphere and the *in vivo* 32 channels hemispherical UF-MEA was placed such that it covered an entire hemisphere of the cortex from prefrontal over motor and somatosensory areas to the visual cortex (see Fig. 3C,D).

#### Data Acquisition

To evaluate the Corticonic board and the UF-MEAs, we directly compared these with conventional products. The recording system was validated through three different hardware configurations, performed *in vitro*. Configuration 1: the conventional 16-channel Pt MEAs (CNM, Barcelona, Spain) as used in previous studies^32^, amplification (Multichannel Systems, Reutlingen, Germany) and AD conversion (Cambridge Electronic Design, Cambridge, UK); Configuration 2: the conventional 16-channel Pt MEAs (CNM, Barcelona, Spain) in combination with the Corticonic board; Configuration 3: the new *in vitro* UF-MEAs in combination with the Corticonic board. In all experimental configurations, data were recorded at a sampling rate of 5 kHz.

#### Stimulation

The stimulation output of the system was validated both *in vivo* and *in vitro*, in order to demonstrate the wide versatility of the system. Particularly, we implemented two different protocols (one *in vivo* and one *in vitro*). In the first protocol, we triggered a LED connected to the stimulation output in order to evoke responses in the visual cortex, which could then be recorded by the system placed on the cortical surface (*in vivo*); In the second, we stimulated cortical slices *in vitro* based on an electric field stimulation protocol^33^. Therefore, external custom-made Ag/AgCl electrodes were connected to the stimulation output of the Corticonic board, and current of different intensities was delivered through the electrodes.

### Data analysis

#### Up state detection

Data analysis was performed with custom-written MATLAB scripts. Up states were detected, as previously described^34^, by setting time and amplitude thresholds in the logarithmically scaled multiunit activity (logMUA). Up state duration and Down state duration is the time from the onset of an Up state to the offset of the same Up state, and the duration of the offset of an Up state to the onset of the following Up state, respectively. The slow oscillation frequency is defined as the inverse of the time from the onset of one Up state to the onset of the following Up state.

#### Spectral signal-to-noise ratio

To evaluate the system quantitatively, we analyzed the signal-to-noise ratio (SNR). The SNR is defined as the ratio of the signal containing meaningful information to the inherent noise of a given recording system. In the case of cortical slow oscillations, the Up state is the signal containing information, while the Down state is expected to be silent and mainly contain noise. Therefore, the spectral SNR is computed as the power of the signal during the Up state divided by the power during the Down state.

$${}_{Spectral}SNR\,=10\ast lo{g}_{10}\frac{\frac{1}{N}{\sum }_{i=1}^{N}{(U{p}_{PSD})}_{i}}{\frac{1}{M}{\sum }_{j=1}^{M}{(Dow{n}_{PSD})}_{j}}[dB],$$

where *N* and *M* are the total number of Up and Down states, respectively, and ${{Up}}_{{PSD}}$ and ${{Down}}_{{PSD}}$ the power spectrum density of the Up and Down states, respectively.

#### Area under the curve of the spectral SNR

The area under the curve (AuC) was computed as the integral of the spectral SNR and was used as an estimator of the SNR in the band of interest. It was calculated as follows:

$$\text{AuC}\,={\int }_{{f}_{0}}^{f}{}_{Spectral}SNR(f)\,df,\,$$

where ${f}_{0}$ and $f$ are the lower and upper frequencies, respectively, for three different frequency bands (low: 5–30 Hz; middle: 30–200 Hz and high: 200–1500 Hz).

#### Voltage SNR

Furthermore, we computed the voltage SNR, which represents the voltage amplitude difference that is observed in the LFP recording at the on- and offset of an Up state. The voltage SNR is based on the ratio between the peak-to-peak (P2P) amplitude of the LFP signal during the Up state and the standard deviation of the signal during the Down state (i.e. the noise):

$${}_{Voltage}SNR=\frac{\frac{1}{N}{\sum }_{i=1}^{N}{(U{p}_{P2P})}_{i}}{\frac{1}{M}{\sum }_{j=1}^{M}{(Dow{n}_{STD})}_{j}},$$

where *N* and *M* are the total number of Up and Down states and *Up*_P2P_ is the peak-to-peak amplitude of the signal during the Up state. ${{Down}}_{{STD}}$ is the standard deviation of the signal during the Down state, computed for a period of one second preceding the Up state.

Finally, the propagation of cortical slow oscillations was computed using an interpolation algorithm on the time lags of onsets of Up states detected in the different electrode pads simultaneously, as proposed by Capone *et al*.^21^.

Statistical differences between two configurations were assessed using the Mann-Whitney U test.

## Results

We first validated the features of the *ad hoc* designed UF-MEAs by evaluating the electrical behavior of the electrodes by electrochemical impedance spectroscopy analysis and by comparing their performance with a previously used conventional 16-channel MEA *in vitro*. Secondly, we validated the Corticonic system (Corticonic board + UF-MEA) by recording slow oscillations *in vitro* and *in vivo*. Finally, we stimulated the cortex and recorded the evoked potential by using both electrical and visual stimulation protocols. The experiments were conceived to capture the propagation of slow oscillations and to demonstrate the capabilities of the Corticonic system to perform closed-loop protocols, which we tested in cortical slices *in vitro*. The experimental setup employed for the tests *in vitro* can be seen in Fig. 3A. In Fig. 3B,C, a detail of different UF-MEA placed onto the neural tissue is shown. Moreover, a general map of the cortex areas of the brain covered by the UF-MEA is depicted in Fig. 3D.

### Electrode arrays

Two designs of UF-MEAs were fabricated for *in vivo* recordings. Both *in vivo* UF-MEAs have 32 recording electrodes distributed homogenously across the device grid to allow the recording of the spatiotemporal evolution (medial to lateral and anterior to posterior) of the cortical activity. The *in vivo* hemispherical UF-MEA was fabricated to cover a hemisphere of the mouse cortical surface and contains 32 electrodes of 50 μm in diameter (Fig. 1Ab), whereas the rectangular UF-MEA was designed with a higher spatial resolution and contains 24 electrodes of 50 µm in diameter, in addition to 2 groups of 4 electrodes with a smaller diameter (10 μm) arranged in a rhomboid (Fig. 1Aa).

Conversely, the *in vitro* UF-MEAs were designed for cortical slices of about 1.5 (depth) × 5 (length) mm^35^ (Fig. 1B). Hence, we designed the *in vitro* UF-MEAs to have 16 electrodes with a diameter of 50 µm distributed over an area of 1 mm vertical by 3 mm horizontal, which is optimal to record neuronal activity across upper (supragranular) and deeper (infragranular) layers in the ferret visual cortex and to study the propagation of cortical slow oscillations (Fig. 3B).

### Impedance of the UF-MEA electrodes

We first measured the impedance of the two different electrode sizes in the UF-MEAs (10 µm and 50 µm in diameter). As expected, the behavior of the electrodes was typically capacitive (Fig. 1C,D). Indeed, the module of impedance decreased almost linearly in a log-log scale as a function of the frequency (Fig. 1C), while the impedance phase stayed close to 80 degrees (Fig. 1D). As expected, the impedance remained higher in the smaller electrodes (Fig. 1C). Furthermore, the electrodes showed a striking homogeneity, as can be seen from the low standard deviation.

### Validation of the Corticonic acquisition board and the UF-MEA

To highlight the potential of the Corticonic system, we compared it with a conventional system. We used the *in vitro* approach of active cortical slices with the aim to test the new system in the most challenging environment. Due to the lower number of neurons in the slice than in the full brain, the network activity (measured as LFP) *in vitro* is usually of lower amplitude than *in vivo* and therefore more sensitive to noise. Furthermore, cortical slices are highly sensitive to changes in the environment, which provided us with an ideal system to test the interaction of the UF-MEA with the neuronal tissue.

Due to the low amplitude of the neuronal signals at the epicortical level (on the surface of the brain), in the range of hundreds of µV, the noise level is a fundamental parameter to describe the efficiency of an acquisition system. Therefore, in order to verify the quality of the acquired signals, we evaluated the noise level and the SNR in 16 cortical slices from three animals.

**Figure 4:**
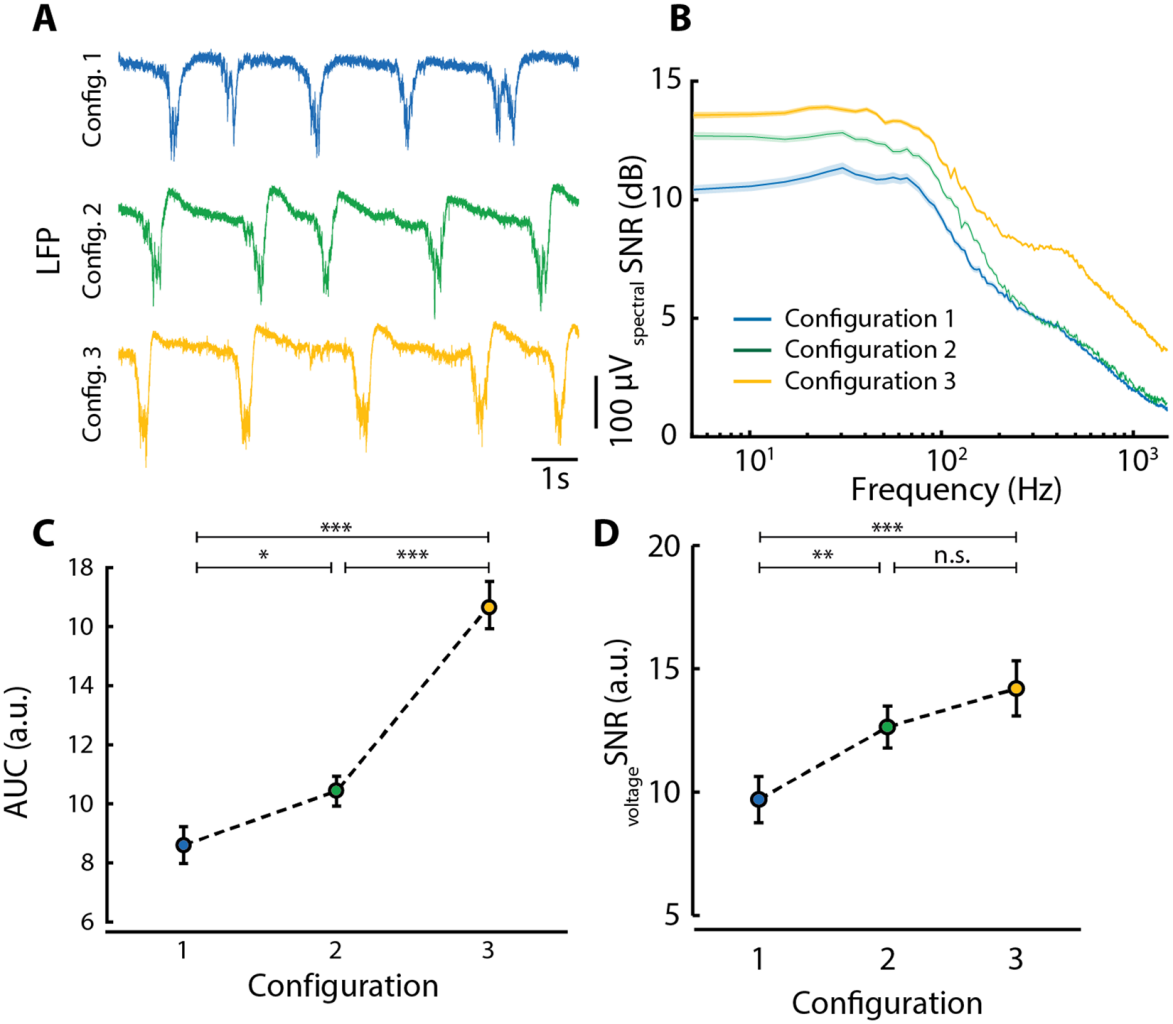
Signal-to-noise ratio (SNR) analysis for the Corticonic system. (**A**) Representative local field potential (LFP) traces recorded during the three different configurations. Configuration 1 (blue): conventional recording system and conventional MEA; Configuration 2 (green): Corticonic recording system + conventional MEA; Configuration 3 (yellow): Corticonic recording system and *in vitro* UF-MEA. (**B**) Averaged spectral SNR across all channels and experiments for the three different configurations across the full frequency spectrum below 1500 Hz. Colored shade: standard error. (**C**) Average of the area under the curve (AuC) of spectral SNR curves below 1500 Hz for the different configurations. (**D**) Average of voltage SNR for the three configurations. C + D: Mann-Whitney U test; *p < 0.05, **p < 0.01, ***p < 0.001; data shown as mean ± standard error.

The raw LFP traces obtained with the three different configurations were similar when performing visual inspection (Fig. 4A). Up and Down states were clearly distinguishable based on the potential difference at the onset and offset of the Up state and the high-frequency content during the Up state. To further compare the systems quantitatively, we performed SNR analysis and took (1) the spectral SNR, (2) the area under the curve (AuC) of the spectral SNR, and (3) the voltage SNR into account.

The spectral SNR was analyzed by inspecting the ratio between the meaningful signal content (i.e. the power during the Up state) and the noise (i.e. the power during the Down state) for the frequency spectrum below 1500 Hz. The neuronal activity captured in LFP recordings is the averaged activity of the local neuronal population (the MUA) and we chose the upper limit of the MUA (1500 Hz) as the maximum of the relevant frequencies. In the given frequency range we observed similar SNR curves shapes for all three configurations (Fig. 4B), but appreciable differences in amplitudes. All spectral SNR curves showed elevated values in the range of 20 to 100 Hz, which is expected physiologically as the neuronal activity synchronizes in high frequencies in the beta and gamma range (15–90 Hz) during the Up state^17,18^. This relative increase in power during the Up state compared to the power during the Down state explains the higher spectral SNR values in this particular frequency range. It is important to note that even if the spectral SNR curves show similar profile the difference of the SNR amplitude indicates a difference in performances among the three configurations in the whole spectrum.

To explain the statistical differences between the three configurations, we used the AuC, which is the area under the spectral SNR curve and gives an estimation of the distribution of spectral SNR values across the frequency spectrum (Fig. 4C). Across the full spectral range below 1500 Hz, we found overall significantly higher AuC values when recording with the Corticonic system (i.e. Config. 3), compared to the conventional system and the conventional MEA (i.e. Config. 1, p = 1.9261^−10^) or the Corticonic board with the conventional MEA (i.e. Config. 2, p = 2.9190^−9^). Actually, the improvement is due to the concurrent better performances of the two parts of the Corticonic system (Board and MEAs), indeed, when the Corticonic board is compared with the conventional system using the same conventional MEA a significant improvement of the SNR is obtained (Config. 1 vs. Config. 2: p = 0.0137). These results indicate that the Corticonic system presents an improved spectral SNR compared to the conventional system.

In addition to the spectral SNR, we analyzed also the voltage SNR, which addresses directly one of the main characteristics of slow oscillations. As Up states present at their onset and offset a prominent, rapid change in the LFP, we used a measurement that compares the three configurations with respect to their performance in recording this feature of slow oscillations. The voltage SNR is computed as the ratio between the peak-to-peak amplitude during the Up state and the standard deviation of the signal during the Down state (i.e. the amplitude of the noise). Therefore, voltage SNR is an ideal parameter to analyze the amplitude of the Up state with respect to the noise during the Down state, independently from the power spectrum. We found higher voltage SNR values for the Corticonic system compared to the conventional system (Config. 1), independent of the used electrode array (MEA, Config. 2, p = 0.0020 or UF-MEA, Config. 3, p = 2.12*10^−4^). In contrast to the spectral SNR, the voltage SNR is solely based on the potential difference between Up and Down state and therefore serves as an estimator in particular for the very low frequencies. Our results on the voltage SNR suggest also in this case a better ratio of Up state amplitude to Down state variability when the recording is performed with the Corticonic system.

**Figure 5:**
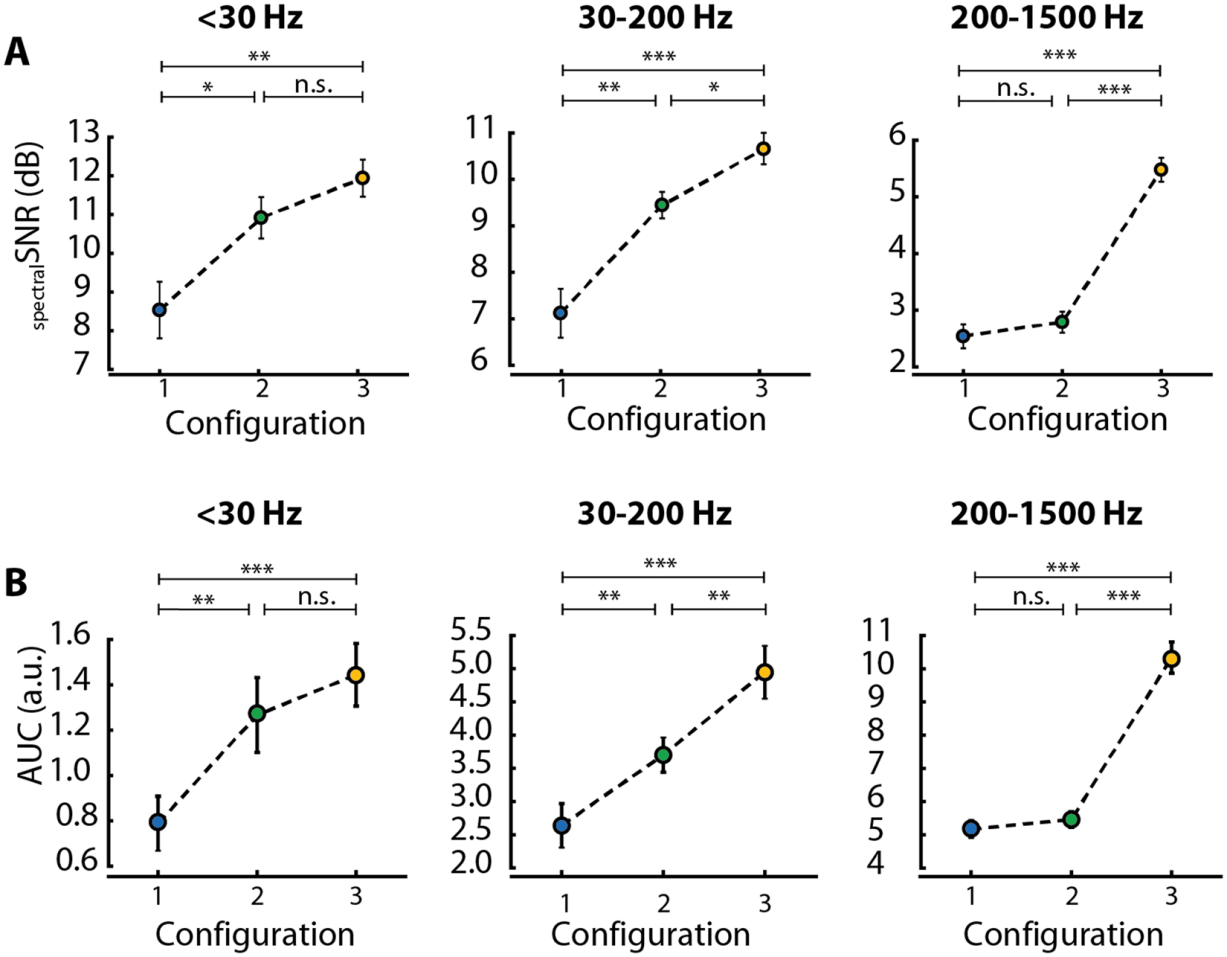
Signal-to-noise ratio analysis for relevant frequency bands. (**A**) Average of spectral SNR values and (**B**) AuC values of all three configurations in three relevant frequency ranges: <30 Hz, left; 30–200 Hz, middle; 200–1500 Hz, right. A + B: Mann-Whitney U test; *p < 0.05, **p < 0.01, ***p < 0.001; data shown as mean ± standard error.

The results presented until now are an unspecific average across the entire frequency spectrum below 1500 Hz. Therefore, in order to point out the potential differences among the configurations for the different frequency bands, we split the spectral SNR and the AuC values among the three frequency bands of interest: (1) < 30 Hz; (2) 30–200 Hz; and (3) the MUA band 200–1500 Hz. For each frequency band, the distribution of spectral SNR values (Fig. 5A) as well as the AuC of the spectral SNR (Fig. 5B) were then computed.

We first analyzed the distribution of spectral SNR values and found that the Corticonic board in combination with the UF-MEAs (Config. 3) showed significantly higher spectral SNR values across all frequency bands (see Fig. 5A). Interestingly, the Corticonic board in combination with the conventional MEA (Config. 2) has a significant higher spectral average SNR values than the conventional system with the conventional MEA (Config. 1) below 200 Hz (<30 Hz: p = 0.0278; 30–200 Hz: p = 0.0026), whereas, it shows a similar behavior in the range 200–1500 Hz. This similar behavior can be connected to intrinsic limitations of the standard MEAs in this frequency range.

In the same way, the distribution of AuC values across the different configurations and frequency bands was similar to the distribution of spectral SNR values. Configuration 3 showed significantly higher spectral SNR values across all frequency bands (see Fig. 5B), and more in detail, spectral SNR showed an improvement of the averaged values from Config. 1 to Config. 2 and Config. 3 in the frequency bands up to 200 Hz (Fig. 5B). In accordance with the findings on spectral SNR values, when recording with the Corticonic board and the conventional MEA (Config. 2) compared to the conventional system (Config. 1) also AuC values show similar behavior (even if a slight improvement is obtained also in this case) in the MUA range.

In conclusion, these results demonstrate that the Corticonic board has a higher SNR and therefore better performance respect to the conventional system. In particular, the system composed of the Corticonic board and the *in vitro* UF-MEAs showed the best SNR values in the whole frequency spectrum of interest. This enhancement of the SNR leads to an improvement in the quality of the acquired signal that could be crucial into detecting new brain activity details.

### Validation of the Corticonic stimulation board

After validating the performance of the systems in acquisition mode, we tested the stimulation board. In particular, we explored two different stimulation protocols to demonstrate the potentialities of the Corticonic system: 1) visually-evoked responses *in vivo*, and 2) constant DC electric field stimulation and recording *in vitro*.

**Figure 6:**
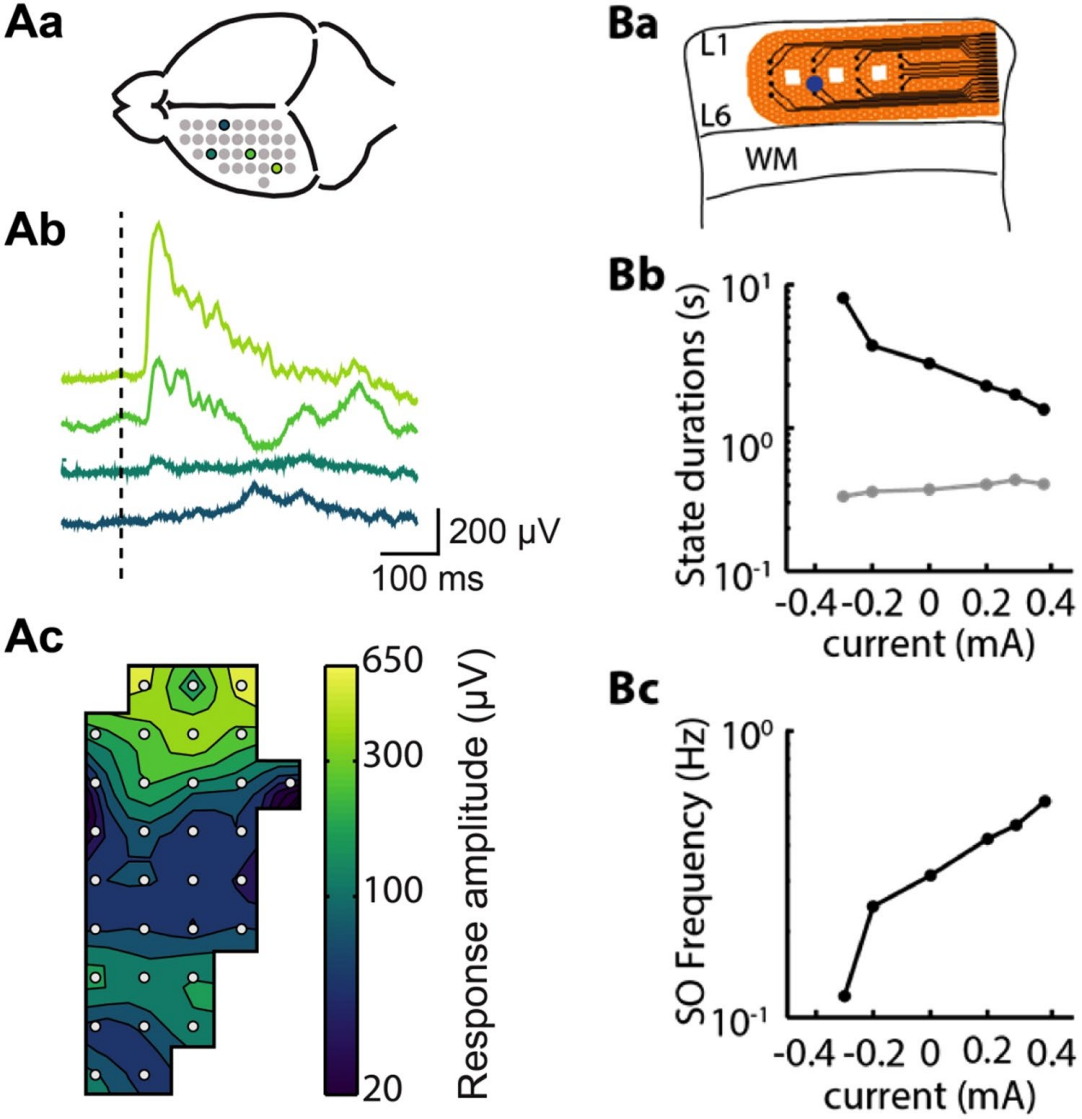
Validation of two different stimulation protocols. (**Aa**) Schema of the UF-MEA placed on the cortical surface in which colored spots represent the locations where the illustrated recordings were obtained (**Ab**) the averaged response (n = 60) to light pulses of 1 ms applied to the contralateral eye in the channels in (**Aa**). (**Ac**) 2D-spline interpolation of the mean response peak in a time window from 0 to 200 ms after the stimulus onset. (**Ba**) Schematic representation of the slice and *in vitro* UF-MEA placement. Blue circle indicates recording site. (**Bb**) Electric field stimulation. Modulation of Up state is shown in gray and Down state duration is shown in black. (**Bc**) The resulting increase in slow oscillation frequency as function of the injected current. WM: White matter; L1 and L6: cortical layer 1 and layer 6, respectively.

In the first experiment, we triggered a LED with the stimulation board (2.5 mA, 0.1 ms) to evoke visual responses in the visual cortex of an anesthetized mouse. The *in vivo* hemispherical UF-MEA was placed on the cortical surface above visual and somatosensory areas (Figs 3C and 6Aa) and the LED was placed in front of the contralateral eye. The averaged visual response on 60 replicas appeared at 40 ms after the LED stimulus and was strongest in electrodes located above the visual area (Fig. 6Ab). Also in this case, we successfully demonstrate the possibility of simultaneously recording and stimulating with the Corticonic board.

Slow oscillation frequency in cortical slices *in vitro* can be modulated with the application of weak DC electric fields^33^. Here, we tested the stimulation output of the Corticonic system by delivering constant current over long time intervals in cortical slices *in vitro* instead of pulsed stimulation used as trigger for the LED *in vivo*. Therefore, we applied, at varying intensities, 60 s of electrical stimulation interposing 20 s of no stimulation, and we found a correlation between increasing current intensities and Down state duration (Fig. 6Bb) that led to an exponential increase in slow oscillation frequency (Fig. 6Bc).

We must point out that the computational power of the digital signal processor (DSP) on the Corticonic board allows performing real time monitoring of the slow oscillation frequency. This feature provided us with the opportunity to integrate a closed-loop algorithm, which performs an online Up state detection and computes the ongoing slow oscillation frequency. This feature was used to implement a closed-loop protocol, which automatically adjusts the intensity of the current output until a desired target frequency is reached.

**Figure 7:**
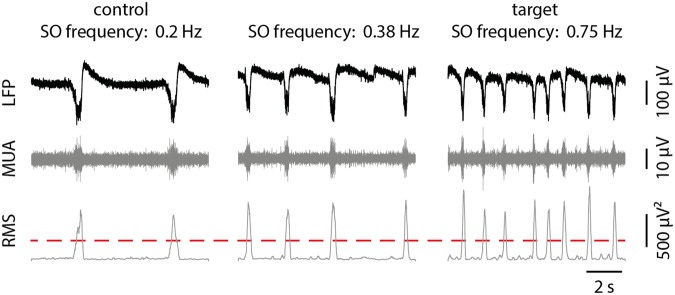
Real-time signal acquisition, filtering and root mean square (RMS) calculation. From left to the right, note the three different time frames with increasing current stimulation, achieving higher SO frequency. Stimulation protocol applied in *in vitro* experiments consisting in a 60 s pulse train stimulation followed by a 20 s pause. The averaging window used to estimate slow oscillation frequency lasts 40 s. By modulating the stimulation current, it is possible to reach the target SO frequency, thus demonstrating the potentialities of the Corticonic system in closed-loop applications. In raw LFP signal traces, several Up states can be observed. In order to estimate the SO frequency, the signal RMS is calculated in the MUA band.

The local computational unit simultaneously executes data filtering, root mean square (RMS) calculation, threshold detection and stimulation tuning. The processes were simultaneously executed with very low latencies (less than 0.5 ms): these values, until now, could not be reached with any other conventional system. The Corticonic board was able to gradually adjust the stimulation output, while constantly monitoring the slow oscillation frequency (computed from the RMS signal), until a specific target frequency was measured (Fig. 7).

These experiments show that the Corticonic system can successfully provide high spatial and temporal accuracy and permits simultaneous stimulation and recording with real-time signal processing.

### Propagation of slow oscillations

**Figure 8:**
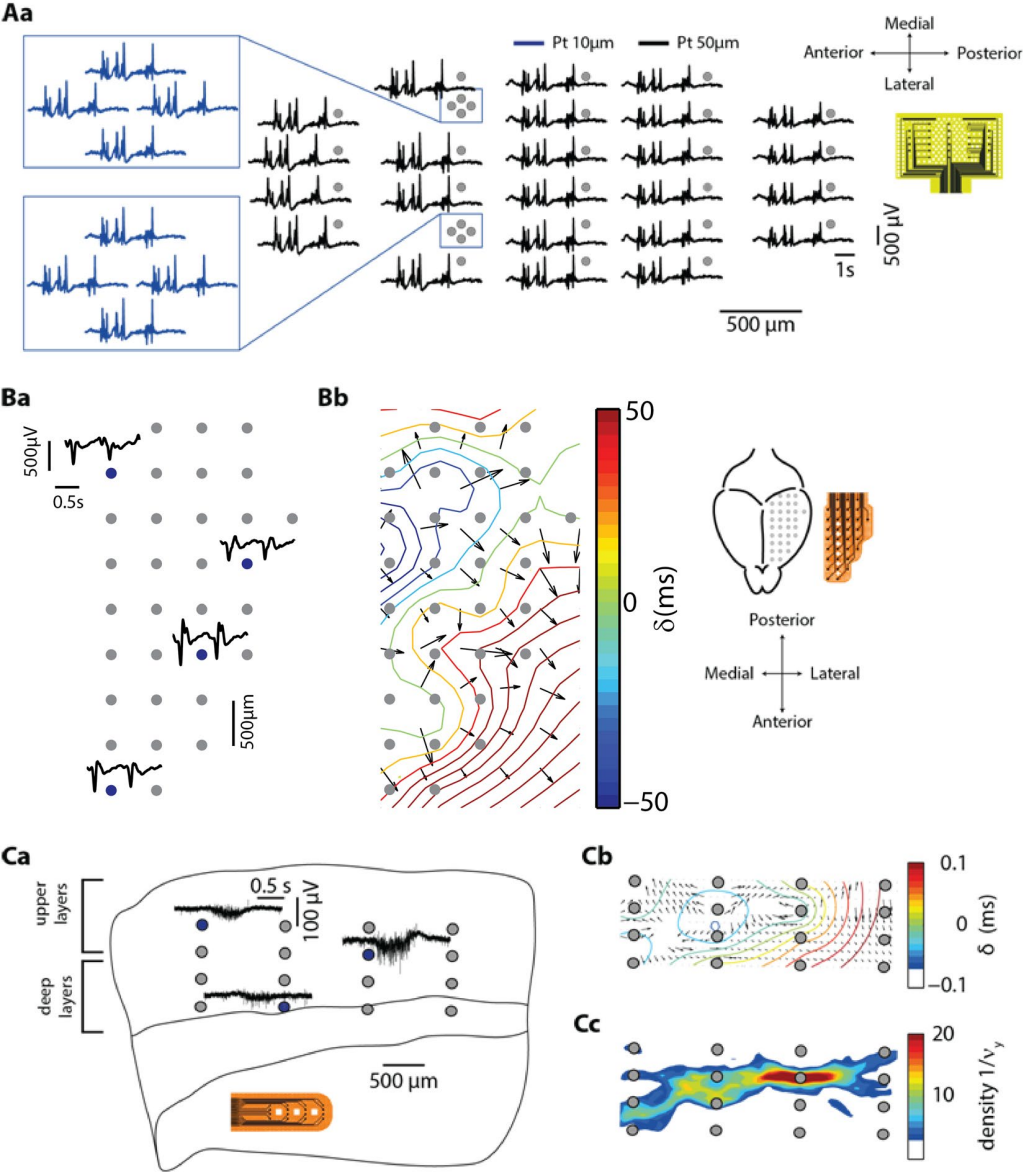
Propagation of slow oscillations measured with the two *in vivo* (**A,B**) and the *in vitro* (**C**) UF-MEA designs. (**Aa**) Map showing 5 s sample traces recorded by each of the 32 electrodes in the *in vivo* rectangular UF-MEA (24 electrodes of 50 µm in diameter and 8 of 10 µm). In the blue inset tone can observe the traces of the 10-µm electrodes. Up states are detected perfectly in all channels, including in the 10-µm electrodes. (**Ba**) Example of traces obtained with the 32-channel *in vivo* hemispherical UF-MEA. (**Bb**) Up states initiated in the midline propagating in various directions covered by the UF-MEA. (**C**) Propagation was also detected with the *in vitro* UF-MEA. (**Ca**) Representative Up state traces at different recording sites in the cortical slice (blue points). (**Cb**) Wave propagation pattern. δ is the mean delay between the detection times of the same wave front at the different pads, it is measured as in Capone *et al*.^36^. (**Cc**) Wave propagation leading area measured as in Capone *et al*.^36^.

Up states are generated locally and they travel as propagating waves throughout the entire cortex or the slice^21^. Here we used a previously described algorithm^36^ to detect wave propagation. Figure 8 shows examples of the propagation recorded with the two *in vivo* UF-MEAs (Fig. 8A,B) and with the *in vitro* UF-MEA (Fig. 8C). The Up states are visible in all channels, including in the 10-µm electrodes. Furthermore, anterior channels recorded more often earlier Up states than did the posterior ones, which is coherent with physiological findings showing stronger neural activity in frontal areas^19^ (Fig. 8A,B). However, note that the wave front did not follow a unique path and may also originate in non-frontal areas (Fig. 8Bb). This is in line with observations obtained from electroencephalographic (EEG) data indicating that Up states can originate everywhere in the cortex and can travel in every direction, although their preferred origin is in frontal areas and their preferred propagation direction is from anterior to posterior. We obtained similar results for recordings with the *in vivo* hemispherical UF-MEA (Fig. 8B).

The propagation of slow oscillations cannot only be observed *in vivo*, but it has also been reported in cortical slices *in vitro*^14,36^ We were able to record propagation of Up states in slices using the Corticonic board and the *in vitro* UF-MEA (Fig. 8C). In agreement with previous findings^19^, the velocity of propagation was slower *in vitro* than *in vivo*; for the shown representative examples: 104 mm/s (Fig. 8A), 76.5 mm/s (Fig. 8B) and 13.1 mm/s (Fig. 8C).

## Conclusions

In this work, we present an ultra-compact integrated system for monitoring and stimulating the cortical network, which we have substantiated here through the recording of cortical slow oscillations. The system is composed of an ultra-flexible microelectrode array (UF-MEA) connected to a compact board (Corticonic board) dedicated to the analysis and modulation of neuronal signals. The system can record and stimulate with low latencies, thus allowing the implementation of closed-loop protocols. Here we used a model of cortical slow oscillations *in vitro* and *in vivo* as test environment.

We successfully validated the data acquisition and stimulation and compared it to a conventional system. The tests prove the Corticonic board’s ability to record slow oscillations with high accuracy. The Corticonic board exhibits good rejection to the 50-Hz power line noise, since the proximity of the acquisition electronics to the signal source enables obtaining better results with respect to conventional systems in terms of signal-to-noise ratio. Moreover, the stimulation board can carry out stimulation protocols of more complex and bulky devices.

Additionally, the combination of recording and stimulation boards coordinated by a digital signal processing on the Corticonic board allows executing real-time data analysis (e.g. MUA filtering and RMS signal calculation) and run specific algorithms during the *in vivo* and *in vitro* tests. These features present the most fundamental prerequisites to realize a closed-loop system and to single out novel slow oscillation patterns in the cortex. Indeed, we measured latencies in data acquisition and processing as low as 0.5 ms while simultaneously recording and stimulating activity *in vitro*. The system has therefore promising applications for *in vivo* closed-loop procedures.

The Corticonic system also successfully identifies the propagation of slow oscillations: it can detect the velocity of propagation and the direction of the traveling waves, and maps these data both *in vivo* and *in vitro* with the possibility to detect signals from small electrode pads (10 µm).

In conclusion, the portable, all-in-one recording and stimulating system that we describe here has proven its potential in the field of neuroelectrophysiology since it allows detecting and manipulating brain activity with very high spatial and temporal accuracy. The results demonstrate that the better performances of the presented device respect to classical laboratory instrumentation can be decisive for new discoveries in electrophysiological research.
